# A pathway linking liver fat and arterial stiffness - direct as well as indirect effects

**DOI:** 10.1186/1532-429X-18-S1-Q39

**Published:** 2016-01-27

**Authors:** Jennifer J Rayner, Rajarshi Banerjee, Jane M Francis, Stefan Neubauer, Oliver Rider

**Affiliations:** grid.4991.50000000419368948University of Oxford, Oxford, United Kingdom

## Background

Although the association of triglycerides (TG) with cardiovascular disease has been debated, accumulating evidence suggests that hypertriglyceridaemia may independently indicate an increased risk of atherosclerotic disease. A subclinical impairment of aortic elastic properties often precedes overt atherosclerotic disease and predicts the corresponding risk. Circulating TG levels are dictated not only by dietary intake but also by hepatic TG production, a process that is elevated in hepatic steatosis. This suggests that hepatic steatosis has indirect effects on vascular stiffness acting through increasing blood TG levels. Using the combination of flow imaging, ^1^H magnetic resonance spectroscopy (MRS) and serum TG level, we investigated the relationship between hepatic fat content, circulating TG levels and aortic pulse wave velocity (PWV) in obesity.

## Methods

65 subjects (male n = 37) across a wide range of body mass index (BMI 18.5-52.6 kg/m^2^) with no identifiable cardiac risk factors (average systolic blood pressure (SBP) 120 ± 12 mmHg, diastolic blood pressure (DBP) 74 ± 7 mmHg, glucose 5.1 ± 0.5 mmol/l, cholesterol 5.1 ± 0.8 mmol/l) underwent ^1^H MRS at 3T to quantify hepatic triglyceride content and flow imaging to assess pulse wave velocity (PWV) between the ascending aorta (pulmonary artery level) and the abdominal aorta.

## Results

PWV was correlated positively with age (r 0.36, p = 0.03), liver fat (r 0.51, p < 0.001) and serum TG (r 0.47, p < 0.001). Multiple regression analysis revealed all three to be independent predictors of increased PWV (TG β 2.2, p = 0.05, liver fat β 2.1, 0.038, age β 0.74, p = 0.042). To further explore the relationship between PWV, liver fat and TG, age adjusted moderated multiple regression was performed with 1000 sample bootstrapping of indirect effects (dependent variable PWV, independent variable hepatic fat, moderator TG). This showed a significant indirect effect of TG on PWV, with 36% of the total negative effect of hepatic steatosis on PWV being attributable to the associated increase in serum TG (p = 0.05). This also showed that 43% of the total negative effect of increased TG on PWV was due to hepatic fat (p < 0.001).

## Conclusions

Increasing age, liver fat and triglycerides are all related to increased aortic stiffness. When controlling for the effects of age, hepatic fat not only has a direct negative effect on pulse wave velocity but also an indirect effect via increased circulating triglyceride level. Of the total negative effect of triglycerides on pulse wave velocity, a significant proportion is related to hepatic steatosis.

**Figure 1:**
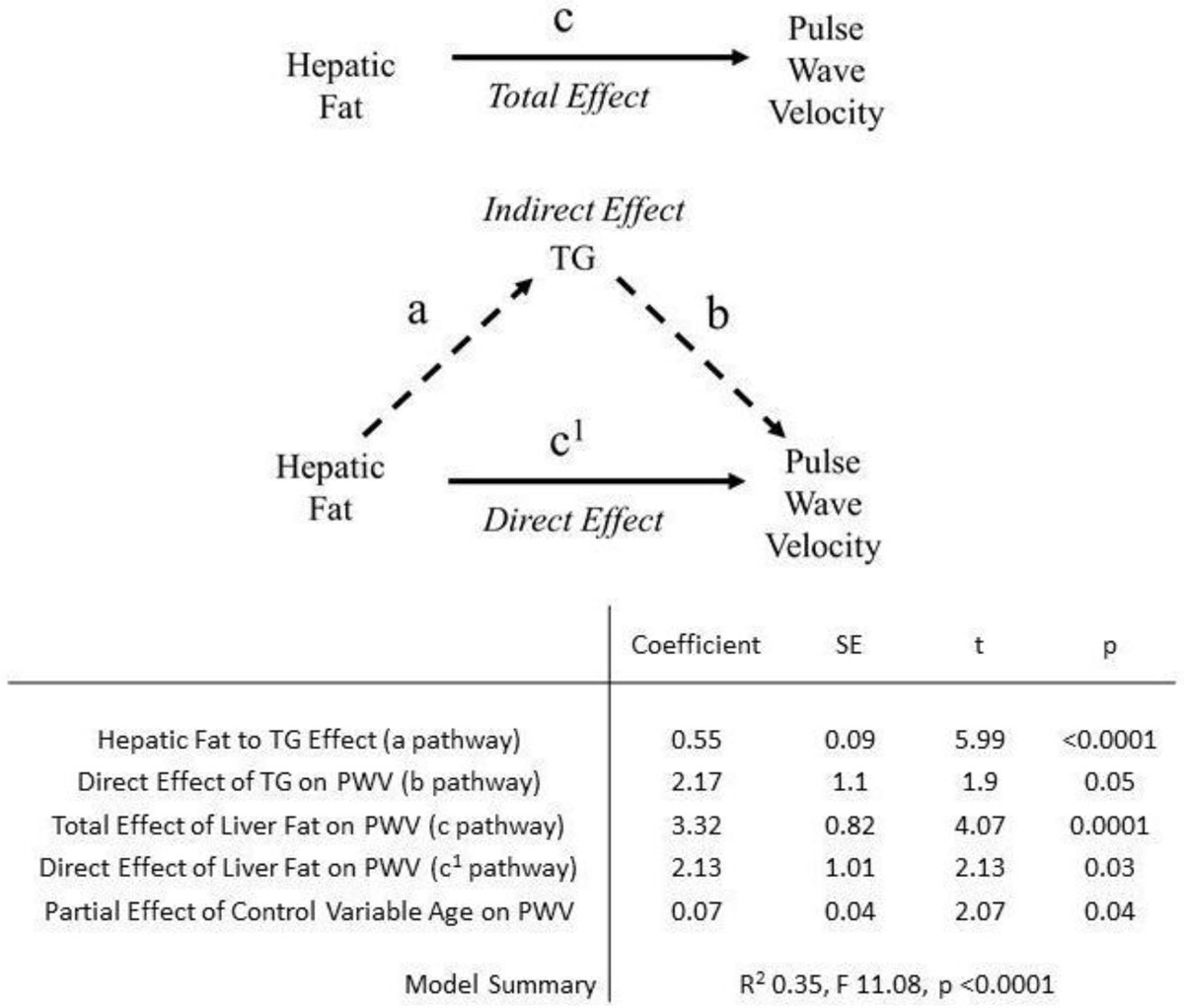
**The contribution of increasing circulating triglycerides (TG) to hepatic steatosis' influence on aortic pulse wave velocity**.

